# Supplementary material for: Therapeutic Efficiency of Proteins Secreted by Glial Progenitor Cells in a Rat Model of Traumatic Brain Injury
Source: Int J Mol Sci. 2023 Aug 2;24(15):12341. doi: 10.3390/ijms241512341 (PMC10419112; doi:10.3390/ijms241512341)
Supplement: Supplementary file 1 [file ijms-24-12341-s001.zip › ijms-2514002-supplementary.pdf]

Table S1. Proteomic analysis of protein complex (Mw 5-100 kDa).

Protein.name	Protein. accession. number	Gene. simbol	GPCs_p15_1	GPCs_p15_2	GPCs_p15_3	Average
T cell receptor alpha joining 56 (Fragment) OS=Homo sapiens OX=966 GN=TRAJ56 PE=4 SV=1	AA75B6Z2	TRAJ56	17.86885	39.22892	23.16522	26.49
Ataxin-1 OS=Homo sapiens OX=966 GN=ATXN1 PE=1 SV=2	P54253	ATXN1	16.13769	3.313253	7.721739	18.58
Isoform 2 of Insulin-like growth factor-binding protein 7 OS=Homo sapiens OX=966 GN=IGFBP7	Q1627	IGFBP7	37.97845	3.566266	1.286957	14.27
Serpin peptidase inhibitor, clade B (Ovalbumin), member 6, isoform CRA_a OS=Homo sapiens OX=966 GN=SERPINB6 PE=3 SV=1	AA24QZX3	SERPINB6	23.73178	0	15.44348	13.58
Annexin OS=Homo sapiens OX=966 GN=ANXA2 PE=2 SV=1	AA24R5Z7	ANXA2	14.23968	3.566266	21.87827	13.23
Filamin-A OS=Homo sapiens OX=966 GN=FLNA PE=1 SV=4	P21333	FLNA	22.78257	0	16.73435	13.18
Desmoglein-1 OS=Homo sapiens OX=966 GN=DSG1 PE=1 SV=2	Q2413	DSG1	7.59417	17.83133	1.295652	11.97
Dermcidin OS=Homo sapiens OX=966 GN=DCD PE=1 SV=2	P8165	DCD	9.492711	16.48193	9.869565	11.52
Serpin peptidase inhibitor, clade E (Nexin, plasminogen activator inhibitor type 1), member 1, isoform CRA_b OS=Homo sapiens OX=966 GN=SERPINE1 PE=3 SV=1	AA24QYT5	SERPINE1	11.39125	0	12.86957	8.87
Filamin-C OS=Homo sapiens OX=966	Q14315	FLNC	17.86885	1.783133	7.721739	8.86

GN=FLNC PE=1 SV=3						
Isoform 2 of Cytosol aminopeptidase OS=Homo sapiens OX=966 GN=LAP3	P28838	LAP3	16.13769	0	1.295652	8.81
Fibronectin OS=Homo sapiens OX=966 GN=FN1 PE=4 SV=1	AA24R462	FN1	2.883965	0	5.147827	8.68
Aspartate aminotransferase, mitochondrial OS=Homo sapiens OX=966 GN=GOT2 PE=1 SV=3	P55	GOT2	11.39125	0	14.15652	8.52
Serpin B3 OS=Homo sapiens OX=966 GN=SERPINB3 PE=1 SV=2	P2958	SERPINB3	4.746356	1.698795	9.869565	8.15
Isoform 1 of Four and a half LIM domains protein 1 OS=Homo sapiens OX=966 GN=FHL1	Q13642	FHL1	14.23968	0	7.721739	7.32
Glutathione synthetase OS=Homo sapiens OX=966 GN=GSS PE=1 SV=1	AA2R8Y43	GSS	14.23968	0	6.434783	6.89
Galectin OS=Homo sapiens OX=966 PE=2 SV=1	AA384MR27	LGALS1	14.23968	0	6.434783	6.89
Gremlin OS=Homo sapiens OX=966 GN=GREM1 PE=2 SV=1	A6XAA7	GREM1	14.23968	0	6.434783	6.89
Parkinson disease protein 7 OS=Homo sapiens OX=966 GN=PARK7 PE=1 SV=2	Q99497	PARK7	15.18834	0	5.147827	6.78
Isoform 4 of Calumenin OS=Homo sapiens OX=966 GN=CALU	O43852	CALU	6.644898	0	11.58269	6.76
Keratinocyte proline-rich protein OS=Homo sapiens OX=966 GN=KPRP PE=1 SV=1	Q5T749	KPRP	0	12.48193	7.721739	6.73
Quinone oxidoreductase PIG3 OS=Homo sapiens OX=966 GN=TP53I3	Q53FA7	TP53I3	12.34525	0	7.721739	6.69

PE=1 SV=2						
Desmoplakin OS=Homo sapiens OX=966 GN=DSP PE=1 SV=3	P15924	DSP	4.746356	7.132531	7.721739	6.53
Polyubiquitin-C (Fragment) OS=Homo sapiens OX=966 GN=UBC PE=1 SV=1	F5H2Z3	UBC	7.59417	3.566266	7.721739	6.29
Isoform 4 of Superoxide dismutase [Mn], mitochondrial OS=Homo sapiens OX=966 GN=SOD2	P4179	SOD2	11.39125	1.783133	5.147827	6.17
Probable aminopeptidase NPEPL1 OS=Homo sapiens OX=966 GN=NPEPL1 PE=1 SV=3	Q8NDH3	NPEPL1	6.644898	0	1.295652	5.65
Prelamin-A/C OS=Homo sapiens OX=966 GN=LMNA PE=1 SV=1	AA6Q8PFJ	LMNA	14.23968	0	2.573913	5.64
Nicotinate-nucleotide pyrophosphorylase [carboxylating] OS=Homo sapiens OX=966 GN=HEL-S-9n PE=1 SV=1	V9HWJ5	HEL-S-9n	12.34525	0	3.868696	5.45
Aspartate aminotransferase OS=Homo sapiens OX=966 PE=2 SV=1	AA14VK69	GOT1	9.492711	0	6.434783	5.39
Glyceraldehyde-3-phosphate dehydrogenase OS=Homo sapiens OX=966 GN=GAPDH PE=1 SV=3	P446	GAPDH	3.797845	5.349398	5.147827	4.76
HBA1 hemoglobin subunit alpha 1 OS=Homo sapiens OX=966 GN=HBA2 PE=3 SV=1	AAK2BMD8	HBA1	4.746356	1.783133	7.721739	4.75
Ferritin OS=Homo sapiens OX=966 GN=FTH1 PE=3 SV=1	AA24R525	FTH1	8.543442	0	5.147827	4.56
Junction plakoglobin OS=Homo sapiens OX=966 GN=JUP PE=3 SV=1	AA24R1X8	JUP	0.949271	7.132531	5.147827	4.50

Cysteine and glycine-rich protein 1 OS=Homo sapiens OX=966 PE=2 SV=1	AA384P5K2	CSRP1	5.695627	0	7.721739	4.47
PRSS3 serine protease 3 OS=Homo sapiens OX=966 GN=PRSS3 PE=2 SV=1	A1A58	PRSS3	2.847813	5.349398	5.147827	4.45
Peroxiredoxin-4 OS=Homo sapiens OX=966 GN=PRDX4 PE=1 SV=1	Q13162	PRDX4	6.644898	0	6.434783	4.36
Acylpyruvase FAHD1, mitochondrial OS=Homo sapiens OX=966 GN=FAHD1 PE=1 SV=2	Q6P587	FAHD1	6.644898	0	6.434783	4.36
Vimentin OS=Homo sapiens OX=966 GN=VIM PE=1 SV=4	P867	VIM	0	1.783133	1.295652	4.26
Ferritin OS=Homo sapiens OX=966 GN=FTL PE=2 SV=1	AA384MDR 3	FTL	8.543442	0	3.868696	4.13
Antithrombin-III OS=Homo sapiens OX=966 GN=SERPINC1 PE=3 SV=1	AA24R944	SERPINC 1	1.441983	0	1.286957	4.00
Isoform 3 of Nucleoside diphosphate kinase B OS=Homo sapiens OX=966 GN=NME2	P22392	NME2	7.59417	1.783133	2.573913	3.98
Cystatin-A OS=Homo sapiens OX=966 GN=CSTA PE=1 SV=1	P14	CSTA	2.847813	0	6.434783	3.94
Thioredoxin OS=Homo sapiens OX=966 GN=TXN PE=2 SV=1	H9ZYJ2	TXN	6.644898	3.566266	1.286957	3.83
Omega-amidase NIT2 OS=Homo sapiens OX=966 GN=NIT2 PE=1 SV=1	Q9NQR4	NIT2	8.543442	0	2.573913	3.76
Isoform 1B of Desmocollin-1 OS=Homo sapiens OX=966 GN=DSC1	Q8554	DSC1	3.797845	3.566266	3.868696	3.74
Fatty acid binding protein 5 (Psoriasis-associated)	E7DVW5	FABP5	5.695627	0	5.147827	3.61

OS=Homo sapiens OX=966 GN=FABP5 PE=3 SV=1						
Protein S1-A8 OS=Homo sapiens OX=966 GN=S1A8 PE=1 SV=1	P519	S1A8	3.797845	1.783133	5.147827	3.58
S-adenosylmethionine synthase OS=Homo sapiens OX=966 PE=2 SV=1	AA14VJP5	MAT2A	6.644898	0	3.868696	3.52
LIM and SH3 domain protein 1 OS=Homo sapiens OX=966 GN=LASP1 PE=4 SV=1	AA24R1S8	LASP1	3.797845	0	6.434783	3.42
Proteasome subunit alpha type OS=Homo sapiens OX=966 GN=HEL-S-276 PE=2 SV=1	AAKK1K4	PSMA7	7.59417	0	2.573913	3.39
Delta-tubulin OS=Homo sapiens OX=966 PE=2 SV=1	B3KML9	TUBB4B	2.847813	1.783133	5.147827	3.26
Protein S1-A6 OS=Homo sapiens OX=966 GN=S1A6 PE=1 SV=1	P673	S1A6	1.898542	3.566266	3.868696	3.19
14-3-3 protein zeta/delta OS=Homo sapiens OX=966 GN=YWHAZ PE=2 SV=1	DPNI1	YWHAZ	5.695627	0	3.868696	3.19
ZYX protein (Fragment) OS=Homo sapiens OX=966 GN=ZYX PE=2 SV=2	B4DQR8	ZYX	5.695627	0	3.868696	3.19
Heat shock cognate 71 kDa protein OS=Homo sapiens OX=966 GN=HSPA8 PE=1 SV=1	P11142	HSPA8	0	1.783133	7.721739	3.17
Protein S1-A7 OS=Homo sapiens OX=966 GN=S1A7 PE=1 SV=4	P31151	S1A7	1.898542	1.783133	2.573913	2.85
Elongation factor 1-alpha 1 OS=Homo sapiens OX=966 PE=4 SV=1	AA4D5RAC 7	EEF1A1	1.898542	1.783133	2.573913	2.85
Neutrophil defensin 1 OS=Homo sapiens OX=966 GN=DEFA1	P59665	DEFA1	2.847813	1.783133	3.868696	2.84

PE=1 SV=1						
Macrophage migration inhibitory factor OS=Homo sapiens OX=966 GN=MIF PE=2 SV=1	I4AY87	MIF	2.847813	1.783133	3.868696	2.84
enolase 1 OS=Homo sapiens OX=966 GN=HEL-S-17 PE=2 SV=1	AA24R4F1	ENO1	1.898542	0	6.434783	2.78
Eukaryotic translation initiation factor 6 OS=Homo sapiens OX=966 GN=EIF6 PE=1 SV=1	P56537	EIF6	5.695627	0	2.573913	2.76
Inositol-1-monophosphatase OS=Homo sapiens OX=966 GN=IMPA1 PE=3 SV=1	AA24R83	IMPA1	5.695627	0	2.573913	2.76
Serpin B12 OS=Homo sapiens OX=966 GN=SERPINB12	Q96P63	SERPINB12	3.797845	1.783133	2.573913	2.72
Lactoylglutathione lyase OS=Homo sapiens OX=966 GN=GLO1 PE=1 SV=4	Q476	GLO1	6.644898	0	1.286957	2.64
Cytochrome c (Fragment) OS=Homo sapiens OX=966 GN=CYCS PE=1 SV=1	C9JFR7	CYCS	0.949271	1.783133	5.147827	2.63
Caspase 14, apoptosis-related cysteine peptidase OS=Homo sapiens OX=966 GN=CASP14 PE=2 SV=1	B2CIS9	CASP14	2.847813	3.566266	1.286957	2.57
Transketolase OS=Homo sapiens OX=966 GN=TKT PE=1 SV=3	P2941	TKT	3.797845	0	3.868696	2.55
transglutaminase 3 OS=Homo sapiens OX=966 PE=3 SV=1	AA494CJ7	TGM3	1.898542	1.783133	3.868696	2.51
Immunoglobulin heavy constant alpha 1 (Fragment) OS=Homo sapiens OX=966	AA286YEY1	IGHA1	1.898542	1.783133	3.868696	2.51

GN=IGHA1 PE=1 SV=1						
Nucleolin OS=Homo sapiens OX=966 GN=NCL PE=1 SV=1	AA7I2V3F3	NCL	4.746356	0	2.573913	2.45
4S ribosomal protein S25 OS=Homo sapiens OX=966 GN=RPS25 PE=1 SV=1	P62851	RPS25	0.949271	3.566266	2.573913	2.36
Myotrophin OS=Homo sapiens OX=966 GN=MTPN PE=1 SV=1	C9JL85	MTPN	5.695627	0	1.286957	2.33
Folate gamma-glutamyl hydrolase OS=Homo sapiens OX=966 GN=GGH PE=1 SV=1	AA7I2YQQ3	GGH	0.949271	0	5.147827	2.32
6S acidic ribosomal protein P2 OS=Homo sapiens OX=966 GN=RPLP2 PE=3 SV=1	AA24RCA7	RPLP2	0.949271	1.783133	3.868696	2.20
Fumarylacetoacetase OS=Homo sapiens OX=966 PE=2 SV=1	AA384P5L6	FAH	3.797845	0	2.573913	2.12
Proteasome subunit beta OS=Homo sapiens OX=966 PE=2 SV=1	AA14VK45	PSMB1	3.797845	0	2.573913	2.12
Myeloid-derived growth factor OS=Homo sapiens OX=966 GN=MYDGF PE=1 SV=1	Q969H8	MYDGF	4.746356	0	1.286957	2.11
C4a anaphylatoxin OS=Homo sapiens OX=966 GN=C4B_2 PE=1 SV=1	AAG2JL54	C4A	0.949271	3.566266	1.286957	1.93
Arginase-1 OS=Homo sapiens OX=966 GN=ARG1 PE=1 SV=2	P589	ARG1	0.949271	3.566266	1.286957	1.93
Cathepsin D OS=Homo sapiens OX=966 GN=CTSD PE=1 SV=1	AA1BGV23	CTSD	1.898542	0	3.868696	1.92
Caldesmon 1, isoform CRA_i OS=Homo sapiens OX=966 GN=CALD1 PE=2 SV=1	AA14VKA	CALD1	1.898542	0	3.868696	1.92
Collagen, type I, alpha 1, isoform CRA_a OS=Homo	D3DTX7	COL1A1	1.898542	0	3.868696	1.92

sapiens OX=966 GN=COL1A1 PE=4 SV=1						
Triosephosphate isomerase OS=Homo sapiens OX=966 PE=2 SV=1	B4DUI5	TPI1	0	1.783133	3.868696	1.88
Peroxiredoxin-1 OS=Homo sapiens OX=966 PE=2 SV=1	AA384NPQ2	PRDX1	2.847813	0	2.573913	1.87
Enhancer of rudimentary homolog OS=Homo sapiens OX=966 GN=ERH PE=3 SV=1	AA24R6D4	ERH	0.949271	1.783133	2.573913	1.77
Fumarylacetoacetate hydrolase domain containing 2A, isoform CRA_a OS=Homo sapiens OX=966 GN=FAHD2A PE=3 SV=1	AA24RE24	FAHD2A	3.797845	0	1.286957	1.69
Proteasome subunit alpha type OS=Homo sapiens OX=966 PE=2 SV=1	AA14VK43	PSMA3	3.797845	0	1.286957	1.69
Peptidyl-prolyl cis-trans isomerase FKBP1A OS=Homo sapiens OX=966 GN=FKBP1A PE=1 SV=2	P62942	FKBP1A	3.797845	0	1.286957	1.69
Coatomer subunit beta' OS=Homo sapiens OX=966 GN=COPB2 PE=1 SV=2	HY938	COPB2	3.797845	0	1.286957	1.69
Kallikrein G OS=Homo sapiens OX=966 GN=KLNG PE=4 SV=1	AA1R3UDR 5	KLK11	1.898542	0	1.286957	1.62
IGKC protein OS=Homo sapiens OX=966 GN=IGK@ PE=1 SV=1	AA5H1ZRQ 3	IGKC	1.898542	0	1.286957	1.62
Heterogeneous nuclear ribonucleoprotein D (AU-rich element RNA binding protein 1, 37kDa), isoform CRA_e OS=Homo sapiens OX=966 GN=HNRPD PE=4 SV=1	AA24RDF4	HNRNPD	1.898542	0	1.286957	1.62
Collagen alpha-1(III) chain OS=Homo sapiens OX=966 GN=COL3A1	P2461	COL3A1	1.898542	0	1.286957	1.62

PE=1 SV=4						
cDNA FLJ51896, highly similar to Glia-derived nexin OS=Homo sapiens OX=966 PE=2 SV=1	B4DMR3	B4DMR3	1.898542	0	1.286957	1.62
colony stimulating factor 2 receptor subunit beta OS=Homo sapiens OX=966 GN=CSF2RB PE=4 SV=1	LR5A1	CSF2RB	0	3.566266	1.286957	1.62
Zinc-alpha-2-glycoprotein OS=Homo sapiens OX=966 PE=2 SV=1	AA14VK	AZGP1	1.898542	0	2.573913	1.50
Lipocalin 1 (Tear prealbumin), isoform CRA_a OS=Homo sapiens OX=966 GN=LCN1 PE=3 SV=1	AA24R8D7	LCN1	1.898542	0	2.573913	1.50
Nucleophosmin (Fragment) OS=Homo sapiens OX=966 GN=NPM1 PE=2 SV=1	AAS2Z491	NPM1	1.898542	0	2.573913	1.50
Gamma-glutamylcyclotransferase OS=Homo sapiens OX=966 GN=C7orf24 PE=4 SV=1	AA9N7V5	GGCT	1.898542	0	2.573913	1.50
Cofilin-1 OS=Homo sapiens OX=966 GN=CFL1 PE=1 SV=3	P23528	CFL1	1.898542	0	2.573913	1.50
Alternative protein RCOR1 OS=Homo sapiens OX=966 GN=RCOR1 PE=4 SV=1	LR8D2	LR8D2	1.898542	0	2.573913	1.50
Transaldolase OS=Homo sapiens OX=966 PE=2 SV=1	AA14VK56	TALDO1	1.898542	0	2.573913	1.50
Reticulocalbin-1 OS=Homo sapiens OX=966 GN=RCN1 PE=1 SV=1	Q15293	RCN1	1.898542	0	2.573913	1.50
Ubiquitin-like protein FUBI; FAU OS=Homo sapiens OX=966 GN=FAU PE=1 SV=1	E9PR3	FAU	1.898542	0	2.573913	1.50

N(4)-(beta-N-acetylglucosaminyl)-L-asparaginase OS=Homo sapiens OX=966 GN=AGA PE=1 SV=2	P2933	AGA	1.898542	0	2.573913	1.50
Non-histone chromosomal protein HMG-17 OS=Homo sapiens OX=966 GN=HMGN2 PE=1 SV=3	P524	HMGN2	1.898542	0	2.573913	1.50
Lactotransferrin (Fragment) OS=Homo sapiens OX=966 PE=2 SV=1	B3KSL2	LTF	0	1.783133	2.573913	1.45
Eukaryotic translation initiation factor 5A (Fragment) OS=Homo sapiens OX=966 GN=EIF5A PE=1 SV=8	I3L397	EIF5A	2.847813	0	1.286957	1.38
Cytochrome c oxidase subunit 7A2, mitochondrial OS=Homo sapiens OX=966 GN=COX7A2 PE=1 SV=1	D6RIE3	COX7A2	0.949271	1.783133	1.286957	1.34
Protein Shroom3 OS=Homo sapiens OX=966 GN=SHROOM3 PE=1 SV=2	Q8TF72	SHROOM3	7.59417	5.349398	18.17391	1.32
Lysozyme C OS=Homo sapiens OX=966 GN=LYZ PE=2 SV=1	B2R4C5	LYZ	0	1.783133	1.286957	1.23
Alpha-2-HS-glycoprotein OS=Homo sapiens OX=966 PE=2 SV=1	B7Z8Q2	AHSG	0	1.783133	1.286957	1.23
ATP synthase membrane subunit K, mitochondrial OS=Homo sapiens OX=966 GN=ATP5MK PE=1 SV=1	Q96IX5	ATP5MK	0	1.783133	1.286957	1.23
Corneodesmosin OS=Homo sapiens OX=966 PE=4 SV=1	AA1U9X8X5	CDSN	0	1.783133	1.286957	1.23
High mobility group AT-hook 1, isoform CRA_b OS=Homo sapiens OX=966 GN=HMGA1	AA24RCT9	HMGA1	1.898542	1.783133	0	1.23

PE=3 SV=1						
ATP synthase subunit alpha, mitochondrial OS=Homo sapiens OX=966 GN=ATP5F1A PE=1 SV=1	P2575	ATP5F1A	0.949271	0	2.573913	1.17
IgG H chain OS=Homo sapiens OX=966 PE=1 SV=1	S6B291	S6B291	0.949271	0	2.573913	1.17
Carboxypeptidase A4 OS=Homo sapiens OX=966 GN=CPA4 PE=2 SV=1	A4D1M3	CPA4	0.949271	0	2.573913	1.17
CD44 antigen OS=Homo sapiens OX=966 GN=CD44 PE=1 SV=3	P167	CD44	0.949271	0	2.573913	1.17
D-aminoacyl-tRNA deacylase OS=Homo sapiens OX=966 GN=DTD1 PE=2 SV=1	Q496C9	DTD1	0.949271	0	2.573913	1.17
Aspartyl aminopeptidase OS=Homo sapiens OX=966 GN=DNPEP PE=3 SV=1	Q53SB6	DNPEP	0.949271	0	2.573913	1.17
Peptidyl-prolyl cis-trans isomerase OS=Homo sapiens OX=966 PE=2 SV=1	A8K486	PPIA	0.949271	0	2.573913	1.17
Apolipoprotein A-I, isoform CRA_a OS=Homo sapiens OX=966 GN=APOA1 PE=2 SV=1	AA24R3E3	APOA1	0.949271	0	2.573913	1.17
Protein S1-A4 OS=Homo sapiens OX=966 GN=S1A4 PE=1 SV=1	P26447	S1A4	0.949271	0	2.573913	1.17
Dynein axonemal heavy chain 12 OS=Homo sapiens OX=966 GN=DNAH12 PE=1 SV=2	E9PG32	DNAH12	0.949271	1.783133	0	0.92
Proteasome subunit beta OS=Homo sapiens OX=966 PE=2 SV=1	E9KL3	PSMB7	0.949271	1.783133	0	0.92
Isoform 3 of Inhibitor of Bruton tyrosine kinase OS=Homo sapiens	Q9P2D	IBTK	0.949271	0	1.286957	0.75

OX=966 GN=IBTK						
Cysteine and glycine-rich protein 2, isoform CRA_a OS=Homo sapiens OX=966 GN=CSRP2 PE=2 SV=1	AA24RBB5	CSRP2	0.949271	0	1.286957	0.75
High mobility group protein 1 OS=Homo sapiens OX=966 GN=HMGB1 PE=3 SV=1	AA24RDR	HMGB1	0.949271	0	1.286957	0.75
cDNA FLJ75914, highly similar to Homo sapiens leucine rich repeat containing 17, mRNA OS=Homo sapiens OX=966 PE=2 SV=1	A8K2P	LRRC17	0.949271	0	1.286957	0.75
Apolipoprotein D (Fragment) OS=Homo sapiens OX=966 GN=APOD PE=1 SV=1	C9JF17	APOD	0.949271	0	1.286957	0.75
Mitochondrial import inner membrane translocase subunit OS=Homo sapiens OX=966 GN=TIMM8B PE=1 SV=1	G3XAN8	TIMM8B	0.949271	0	1.286957	0.75
Oxygen-regulated protein 1 OS=Homo sapiens OX=966 GN=RP1 PE=1 SV=1	AA1BGTV9	RP1	0.949271	0	1.286957	0.75
cDNA FLJ53619, highly similar to Heat shock protein HSP 9-beta OS=Homo sapiens OX=966 PE=2 SV=1	B4DGL	B4DGL	0.949271	0	1.286957	0.75
Tropomyosin 1 (Alpha) isoform 4 OS=Homo sapiens OX=966 GN=TPM1 PE=3 SV=1	D9YZV5	TPM1	0.949271	0	1.286957	0.75
Heterogeneous nuclear ribonucleoproteins A2/B1 OS=Homo sapiens OX=966 GN=HNRNPA2B1 PE=1 SV=1	AA7I2V4I6	HNRNPA 2B1	0.949271	0	1.286957	0.75
NAD(P)H dehydrogenase, quinone 2, isoform CRA_a OS=Homo sapiens	AA24QZT9	NQO2	0.949271	0	1.286957	0.75

OX=966 GN=NQO2 PE=3 SV=1						
NIF3-like protein 1 OS=Homo sapiens OX=966 GN=NIF3L1 PE=3 SV=1	AA24R3V5	NIF3L1	0.949271	0	1.286957	0.75
Annexin A5 OS=Homo sapiens OX=966 GN=ANXA5 PE=1 SV=2	P8758	ANXA5	0.949271	0	1.286957	0.75
Beta-glucuronidase OS=Homo sapiens OX=966 GN=GUSB PE=1 SV=2	P8236	GUSB	0.949271	0	1.286957	0.75
Ferric-chelate reductase 1 OS=Homo sapiens OX=966 GN=FRRS1 PE=2 SV=2	Q6ZNA5	FRRS1	0.949271	0	1.286957	0.75
Extracellular glycoprotein lacritin OS=Homo sapiens OX=966 GN=LACRT PE=1 SV=1	Q9GZZ8	LACRT	0.949271	0	1.286957	0.75

Table S2. Primer sequences used for gene expression analysis by RT-PCR.

Gene	Primer sequences	Annealing temperature, °C
Mmp2	for CAGACAAAGAGTTGGCAGTG<br>rev TCAGGTGTGTAACCAATGATC	56.5
Mmp9	for ATGGTTTCTGCCCCAGTGAG<br>rev CACCAGCGATAACCATCCGA	63
Ttnfa	for CCACCACGCTCTTCTGTCTA<br>rev GCTACGGGCTTGTCACTCG	61.9
Gapdh	for GAGATTACTGCCCTGGCTCC<br>rev GCTCAGTAACAGTCCGCCTA	56.65
Actb	for GCGAGATCCCGCTAACATCA<br>rev CCCTTCCACGATGCCAAAGT	56
Il12a	for CTGCCAAGTGTCTTAACCAGT<br>rev GCAGGCCTCCAGTGTGCT	60.5
Il12b	for CTGGTGTCTCCACTCATGG<br>rev CAGGTGTATTGGCCAGCATC	60.5
Il1b	for CTGTCTGACCCATGTGAGCT<br>rev ACTCCACTTTGGTCTTGACTT	57.4
Il6	for TACATATGTTCTCAGGGAGAT<br>rev GGTAGAAACGGAACTCCAG	56
Il10	for GCCCAGAAATCAAGGAGCAT<br>rev TGAGTGTCACGTAGGCTTCTA	58.8

Timp1	for CAGACAGCTTTCTGCAACTC<br>rev CACAGCGTCGAATCCTTTGA	58.8
Timp2	for ATGCAGACGTAGTGATCAGG<br>rev AGTCACAGAGGGTAATGTGC	56
Bax	for TTGTGGCTGGAGTCCTCACT<br>rev TTTCCCCGTTCCCCATTCATC	63
Bcl2	for GGGGCTACGAGTGGGATACT<br>rev GACGGTAGCGACGAGAGAAG	62.6
Il4	for ATGTAACGACAGCCCTCTGA<br>rev AGCACGGAGGTACATCACG	56.7
Il23	for AGAGGGTCTAGTGCAGAGCA<br>rev ATACGGGGCACGTCACTTTT	59.4
Il18	for GACAAAAGAAACCCGCCTG<br>rev ACATCCTTCCATCCTTCACAG	61.5

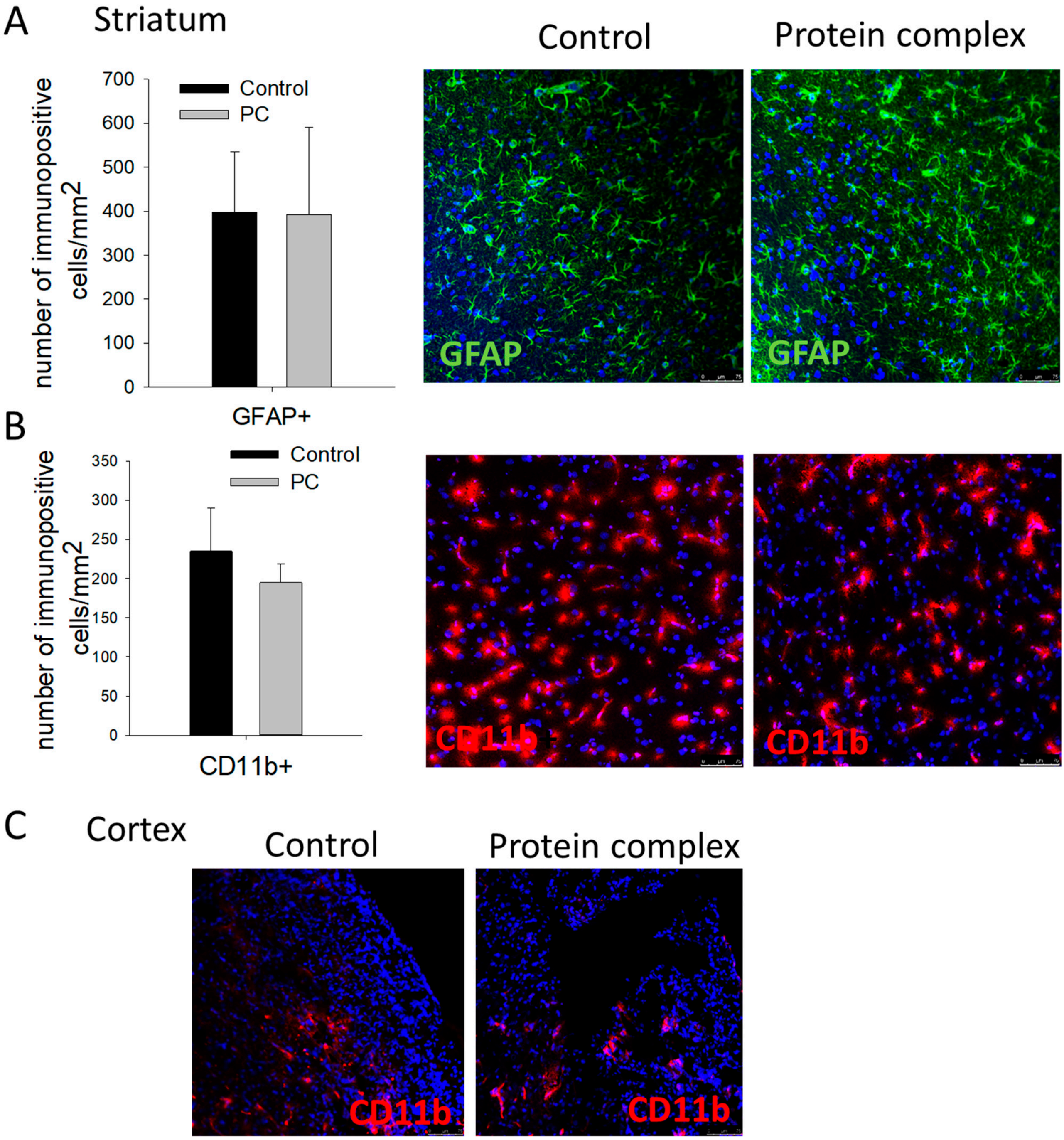

Figure S1. Immunohistochemical analysis of brain tissues (cortex, striatum) on day 14. Visualization and quantitation of astroglia (GFAP+) (**A**), and microglial cells (CD11b+) (**B**) in the striatum; cell nuclei counterstained with DAPI (blue);. **(C)** The single microglial cells (CD11b+) at the cortex damage area. Scale bars, 75 µm. The data presented as means ± SD, and analyzed by t-test or Mann–Whitney test.

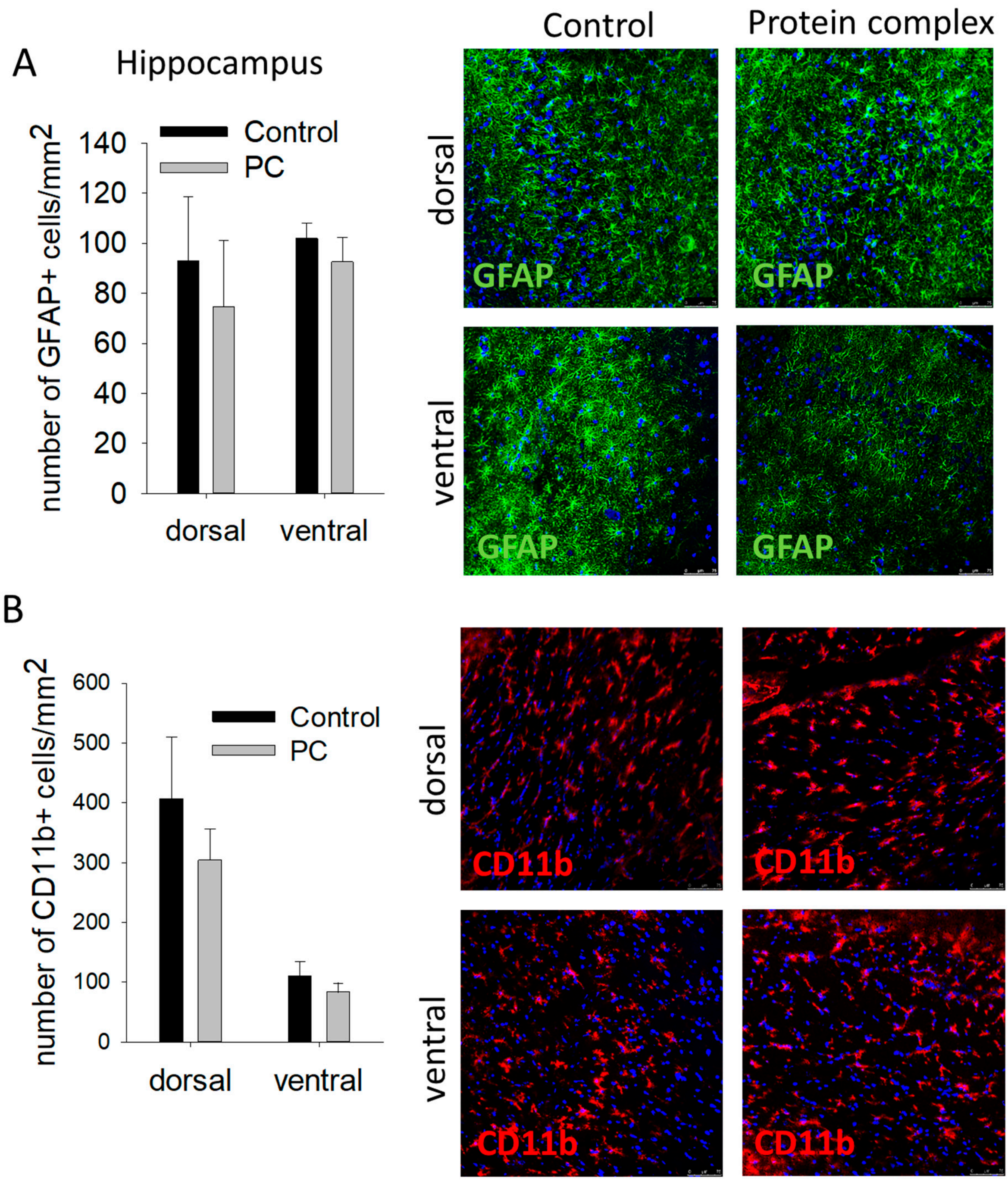

Figure S2. Immunohistochemical study of brain tissues (hippocampus) on day 14. Visualization and quantitation of astroglia (GFAP+) (**A**), and microglial cells (CD11b+) (**B**) in the hippocampus; cell nuclei counterstained with DAPI (blue); scale bars, 75 µm. The data presented as means ± SD, and analyzed by t-test or Mann–Whitney test.

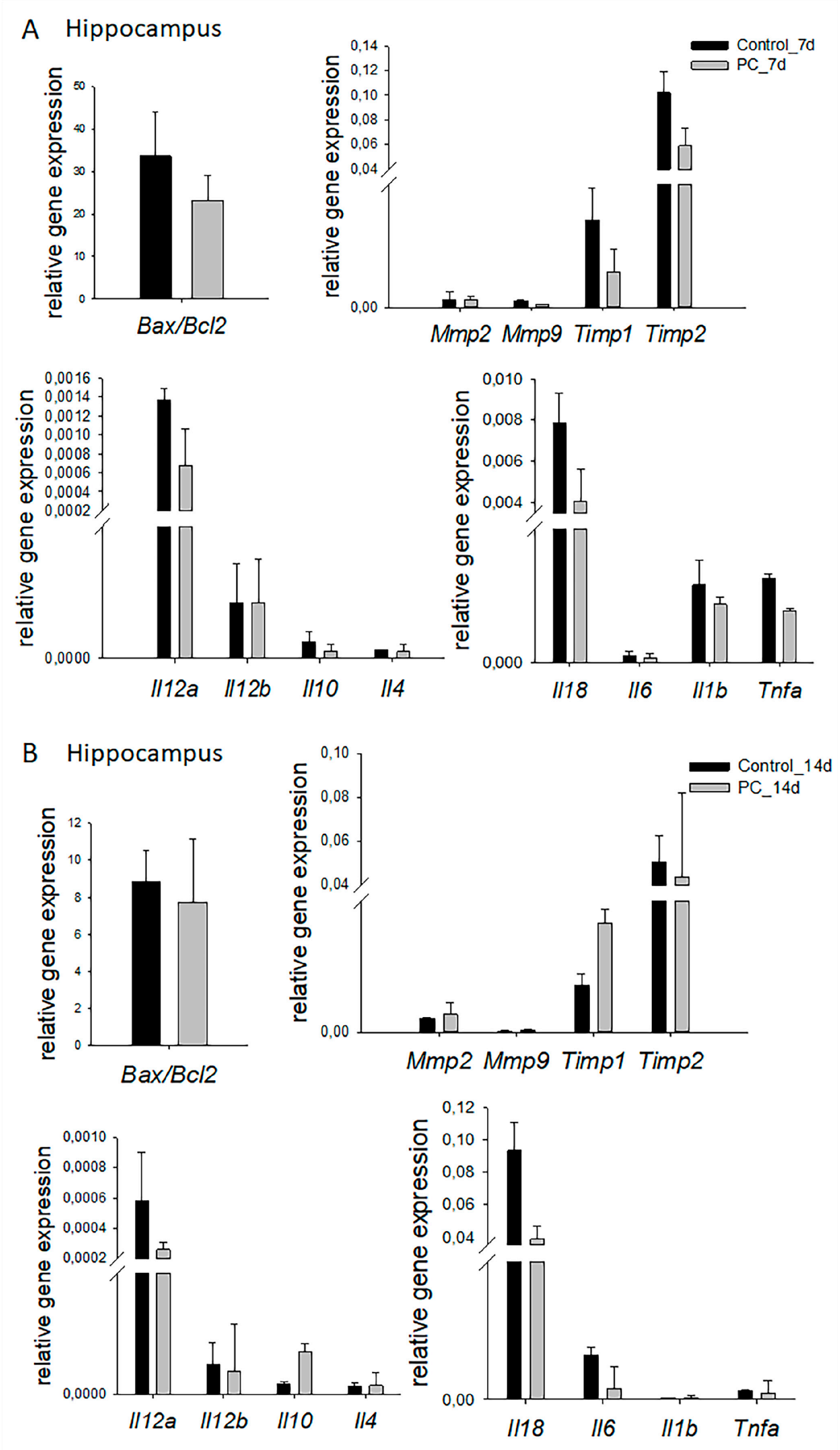

Figure S3. Relative expression levels of apoptosis-, extracellular matrix degradation- and inflammation-related genes in hippocampus on days 7 (**A**) and 14 (**B**). RT-PCR. The data presented as means ± SD, analyzed by *t*-test or Mann–Whitney test.

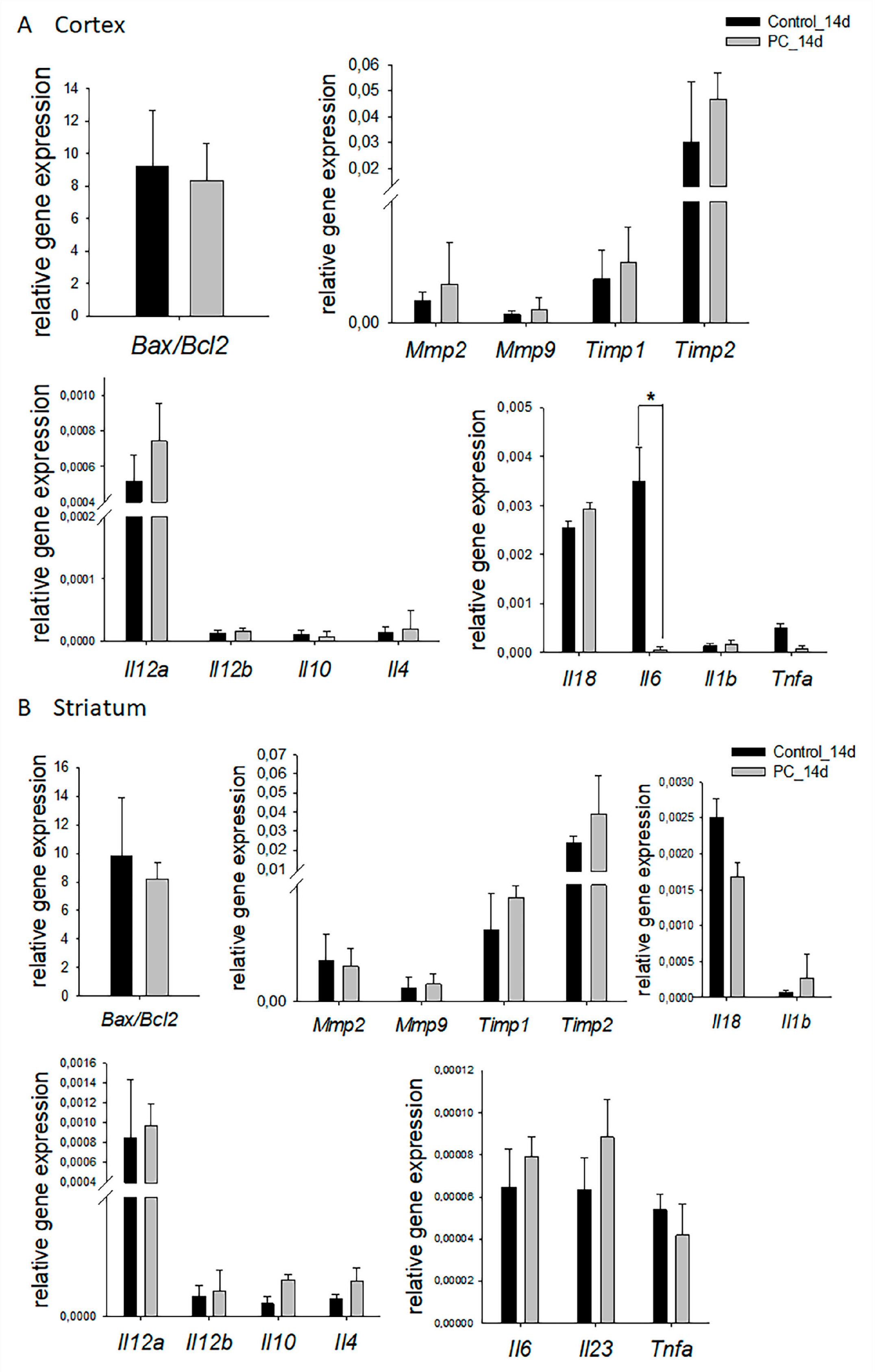

Figure S4. Relative expression levels of apoptosis-, extracellular matrix degradation- and inflammation-related genes in cortex (**A**) and striatum (**B**) on day 14. RT-PCR. The data presented as means ± SD, and analyzed by *t*-test or Mann–Whitney test; * — $p<0.05$ compared with the control.
